# Effect of Physician-Delivered Patient Education on the Quality of Bowel Preparation for Screening Colonoscopy

**DOI:** 10.1155/2013/570180

**Published:** 2013-12-17

**Authors:** Tze-Yu Shieh, Ming-Jen Chen, Chen-Wang Chang, Chien-Yuan Hung, Kuang-Chun Hu, Yang-Che Kuo, Shou-Chuan Shih, Horng-Yuan Wang

**Affiliations:** ^1^Division of Gastroenterology, Department of Internal Medicine, Mackay Memorial Hospital, Taipei, Taiwan; ^2^Mackay Junior College of Medicine, Nursing and Management, Taipei, Taiwan; ^3^Mackay Medical College, New Taipei, Taiwan; ^4^Health Evaluation Center, Mackay Memorial Hospital, Taipei, Taiwan

## Abstract

*Background*. Inadequate bowel preparation is common in outpatients undergoing screening colonoscopy because of unawareness and poor adherence to instruction. *Methods*. Herein, 105 consecutive outpatients referred for screening colonoscopy were enrolled in this prospective, colonoscopist-blinded study. The patients were assigned to an intensive-education group, with 10 minutes of physician-delivered education, or to standard care. At the time of colonoscopy, the quality of bowel preparation was assessed using the Boston Bowel Preparation Scale (BBPS). The primary outcome was a BBPS score ≥5. The secondary outcomes were the mean BBPS score, insertion time, adenoma detection rate, and number of adenomas detected. *Results*. We analyzed 39 patients who received intensive education and 60 controls. The percentage of adequate bowel preparations with a BBPS score ≥5 was higher in the intensive-education group than in the control group (97.4% versus 80.0%; *P* = 0.01). The adjusted odds ratio for having a BBPS score ≥5 in the intensive-education group was 10.2 (95% confidence interval = 1.23–84.3; *P* = 0.03). Other secondary outcomes were similar in the 2 groups. *Conclusions*. Physician-delivered education consisting of a brief counseling session in addition to written instructions improves the quality of bowel preparation in outpatients undergoing screening colonoscopy.

## 1. Introduction

According to the US National Polyp Study, colorectal cancer can be prevented by colonoscopic removal of adenomatous polyps; the long-term survey confirmed that polypectomy could lower colorectal cancer death rates [1]. Successful colonoscopic screening and polypectomy rely on adequate bowel preparation. However, inadequate bowel cleanliness has been reported in up to 30% of patients undergoing colonoscopy [2], making the procedure difficult and timeconsuming and resulting in missed lesions (22–48%) [3, 4], increased risk of complications, the need for repeat examinations, and a 9–22% increase in expenditure [5, 6]. Many factors affect the quality of preparation, including the cleansing agent used, method of purgative administration (e.g., single dose versus split dose) [7], time interval between bowel preparation, start of colonoscopy [8], and appointment waiting time [9]. Some factors are also related to patient characteristics such as inpatient status [2, 10], presence of comorbidities [10], low education level [9], and compliance with bowel preparation instruction [2, 10, 11].

Improving patient understanding of the rationale for bowel preparation before colonoscopy might enhance adherence to the prescribed bowel regimen. However, patient education is an underappreciated element of colonoscopy preparation. The literature contains several reports on the effect of patient education administered by various healthcare professionals on the quality of bowel preparation, but we know of none on the effects of physician-oriented patient education on bowel preparation for screening colonoscopy. We hypothesized that physicians giving outpatients a direct counseling session would enhance patients' adherence to instruction and consequently improve the quality of bowel preparation.

## 2. Methods

### 2.1. Patients and Bowel Preparation

Consecutive patients scheduled for colonoscopy for cancer screening at outpatient clinics were prospectively enrolled. All enrolled patients were visited by one of three physicians *with their specialty of gastroenterology*, who reviewed their medical history and scheduled their colonoscopy. The patients were assigned to either the control group (those who visited 2 outpatient physicians) or the intensive-education group (those who visited 1 index outpatient physician) according to which physician they visited. All patients received a split-dose oral sodium phosphate solution (Fleet Phospho-soda, C.B. Fleet Co., Inc., Lynchburg, VA, USA). The split doses were divided into 2 portions: 45 mL sodium phosphate solution with more than 1500 mL water administered in the evening (8 p.m.) before the examination and a second dose of 45 mL followed by 1500 mL water on the morning (8 a.m.) of the examination. The colonoscopy procedure was scheduled between 2 and 5 p.m.

### 2.2. Instructions

Existing written instructions at our hospital include how to take the oral sodium phosphate solution, with illustrations of the type of food allowed before colonoscopy, and colonoscopic views of good and poor bowel preparation. This information is provided by outpatient nurses at the visit when the colonoscopies are scheduled. In this study, the intensive-education group received the same written instruction. However, in addition, they received a 10-minute counseling session by the index physician at the same visit to discuss the importance of bowel preparation and how the preparation solution should be taken. The counseling emphasized 3 points: proper diet before colonoscopy, adequate hydration with the bowel preparation regimen, and the right times to ingest the purgative. In order to address the importance and rationale of bowel preparation, we addressed 2 opposite points: inadequate bowel preparation may lead to missed lesions, and good bowel preparation favors a good outcome and prevention of cancer. The Institutional Review Board of Mackay Memorial Hospital approved the study, and we obtained written informed consent from all the participants.

### 2.3. Colonoscopic Procedure and Assessment

A single experienced colonoscopist, blinded to the patients' instruction group, performed the procedures. The entire colon was initially examined in a standard manner with conventional white-light endoscopy. The insertion time was defined as the interval between the start of the procedure and arrival at the cecum, with identification of the appendiceal orifice. After removing excess colonic content by suction, the endoscopist was free to use as many flushes as deemed necessary to permit a satisfactory view of the mucosa. A record was kept of the insertion time, number of polyps detected, and location of the polyps. The quality of bowel preparation was graded by use of the Boston Bowel Preparation Scale (BBPS), which is a valid and reliable measure of bowel preparation [12]. Briefly, a 4-point scoring system is applied to each of 3 broad regions of the colon: right side (the cecum and ascending colon), transverse section (from the hepatic flexure to the splenic flexures), and left side (the descending colon, sigmoid colon, and rectum). Points (segment score) were assigned as follows: unprepared colon segment, 0; major residual stool or opaque liquid, 1; minor residual staining, 2; and entire mucosa easily visible, 3. Thus, the maximum BBPS score for a perfectly clean colon is 9, and the minimum BBPS score for an unprepared colon is 0. The scores of each patient were assigned by the colonoscopist, who was blinded to the preparation instructions given. Previous validation studies have shown that a BBPS score of ≥5 is associated with a higher polyp detection rate and is considered adequate bowel preparation [11]. We performed a biopsy of or removed all polyps identified. We counted the number of adenomatous polyps detected during the colonoscopies, and their histologic type was confirmed by histopathological examination.

Our primary endpoint was adequate bowel preparation, that is, a BBPS score ≥5. The secondary outcomes were the mean BBPS score, insertion time, adenoma detection rate, and number of adenomas detected.

### 2.4. Statistical Analysis

Descriptive statistics for continuous data were calculated and were reported as mean ± standard deviation. Categorical variables were described using frequency distributions and were reported as *n* (%). Baseline characteristics and assessment results for the education group and controls were evaluated using Student's *t*-test for continuous variables and chi-square test for categorical variables. A *P* value <0.05 was considered statistically significant.

A logistic regression model was used for analyzing BBPS scores ≥5 for the education and control groups. Regressions with a generalized linear model were also performed to examine the relationship between education and the BBPS score. The *β*-value and 95% confidence interval (95% CI) were calculated. All statistical analyses were conducted using the SAS software, version 9.2. (SAS institute, Inc. Cary, NC, USA).

## 3. Results

### 3.1. Demographic Data and Exclusion Criteria

In this study, 105 patients were prospectively enrolled. Because the appointment colonoscopy waiting times influence the quality of bowel preparation [8], we excluded 2 patients whose appointments were more than 16 weeks after their procedure was scheduled. In addition, 4 patients in the control group were excluded because of their intolerance for the procedure (one patient), technical difficulties (two patients), or active lower gastrointestinal bleeding (one patient). The remaining 99 patients (39 patients in the intensive-education group and 60 patients in the control group) were included in the study. Twenty-five patients (64.1%) in the intensive-education group and 37 (61.7%) in the control group were men. The mean age of patients in the intensive-education group was 46.1 ± 10.9 years, and in the control group was 52.8 ± 14.3 years (Table 1).

### 3.2. Bowel Preparation according to the Mean BBPS Score, Insertion Time, Polyp Detection Rate, and Number of Polyps Detected

The colonoscope insertion time was similar in both groups: 8.7 minutes in the intensive-education group and 9.2 minutes in the control group. The proportion of bowel preparations with a BBPS score ≥5 differed significantly between the 2 groups: 97.4% in the intensive-education group versus 80% in the control group (*P* = 0.01). In addition, the mean BBPS score was significantly different in the 2 groups: 7.3 ± 1.4 in the intensive-education group and 6.4 ± 1.9 in the control group (*P* = 0.012). The segment score of the transverse colon region was 2.6 ± 0.5 in the intensive-education group and 2.2 ± 0.8 in the control group (*P* = 0.006), and the score of the left side was 2.7 ± 0.4 in the intensive-education group and 2.4 ± 0.6 in the control group (*P* = 0.001). The score of right side was not significantly different. The adenoma detection rate and the number of polyps detected were higher in the intensive-education group as compared with the control group, but the differences were not statistically significant (polyp detection rate, 37.5% versus 20%, *P* = 0.17; number of polyps detected, 38.5% versus 22.0%, *P* = 0.07) (Table 1).

Because the difference in mean age of the subjects in each group could be a confounding factor, the data were further analyzed by a logistic regression model to adjust for age and sex; the adjusted odds ratio for having a BBPS score ≥5 in the intensive-education group was 10.2 (95% CI = 1.23–84.3; *P* = 0.03) compared with controls. In the generalized linear regression model, there was no significant difference in mean BBPS score between the education and control groups (*β* = 0.96; 95% CI = 0.25, 1.67; *P* = 0.21). There were differences between the 2 groups for segment scores of the transverse colon (*β* = 0.42; 95% CI = 0.13, 0.70; *P* = 0.045) and left side of the colon (*β* = 0.27; 95% CI = 0.06, 0.49; *P* = 0.01). However, there was no significant difference in the scores of the right side of the colon (*β* = 0.21; 95% CI = 0.10, −0.52; *P* = 0.18).

## 4. Discussion

In our physician-delivered education program, a brief counseling session in addition to the usual written instructions improved the quality of bowel preparation in outpatients undergoing screening colonoscopy. In a reported study, the authors felt that suboptimal bowel preparation resulted from patients' lack of appreciation of the importance of the preparation, lack of confidence in ability to follow the instructions, and confusion about the precolonoscopy diet [13]. Optimal results from colonoscopy preparation have been observed when the sessions are conducted in a comfortable setting without interruption and when communication with the education provider is good [14]. We suggest that improvement in patient understanding of the rationale for bowel preparation as well might enhance adherence to bowel cleaning regimens and accordingly improve the quality of bowel preparation.

Reported efforts to improve the effectiveness of colonoscopy bowel preparation have included cartoon visual aids, educational booklets, brief counseling sessions, questionnaires, interactive voice-response systems to ensure that patients attend appointments [15], and telephone reeducation to notify patients on the day before colonoscopy [16]. However, results with these efforts have been inconsistent.

Nurse-delivered education with brochures [15], instructions plus educational pamphlets sent by mail 3 weeks before the procedure [17], novel patient educational booklets [18], education with cartoon visual aids organized by the health examination center staff [19], and telephone reeducation about the details of bowel preparation on the day before colonoscopy by a physician [16] reportedly can improve the quality of bowel preparation. However, other interventions, such as mailed photographs of adequate or inadequate colons [20] or a question-and-answer session by senior gastroenterology fellows [21] have failed to improve bowel preparation quality.

When the instructions are administered by mail, they may not adequately explain the procedure, and the patients may not understand the message. Providing patients with both oral and written instructions for bowel preparation may be more effective than written instructions only. The European Society of Gastrointestinal Endoscopy recommends that oral and written information about bowel preparation be delivered together by healthcare professionals [22]. However, the education level of healthcare professionals, such as nurses, gastroenterology fellows, or visiting physicians, may influence the education of the patient. For example, one study of 164 patients showed no difference in the quality of bowel preparation when standard instructions plus a questionnaire and a face-to-face meeting with a gastroenterology fellow were used [21]. Perhaps patients lack confidence in the education provided by a gastroenterology fellow. Whether results of such studies can be generalized to other education providers is not known.

In our study, we defined the threshold for an adequate bowel preparation as a BBPS score ≥5. In a previous study of 633 screening colonoscopies, a BBPS score ≥5 was associated with a higher polyp detection rate (40% versus 24%) [11]. We applied these BBPS measures of the quality of preparation after cleansing maneuvers when washing and suctioning of fluid have been completed. This approach is more clinically relevant for determining the likelihood of missed lesions than just assessment of the method of colonic preparation when the bowel is not adequately distended without suction or flushing procedures. Our study showed that 97% of the intensive-education group had BBPS scores ≥5; a similar rate (96.7%) was achieved in a large colonoscopy project carried out in Berlin [23].

Because there was a difference in age between the 2 groups, which may have been be a confounding factor, we performed analysis using a generalized linear regression model. Consequently, we identified differences between the study groups in the segment scores for the transverse and left colon (*P* = 0.01), but no difference for scores of the right colon. This result may reflect the findings of some studies, wherein colonoscopy was found to be less effective in preventing cancers in the proximal colon than in the distal colon [24], a difference that may reflect the difficulty in cleansing the right colon even after efforts towards improving patient education. It has been reported that polyethylene glycol may be superior to sodium phosphate in cleansing the right colon (odds ratio = 2.36; 95% CI = 1.16–4.77; *P* = 0.012) [24]; verification of this observation in the setting of intensive patient education is warranted.

Our study has notable strengths. First, it is a single colonoscopist-blinded, prospective trial; this approach assured uniformity in the washing and suctioning of residual material from the colon during the colonoscopy. Second, we controlled for factors known to influence bowel preparation quality, such as the cleansing agent used, timing of purgative administration, and interval between bowel preparation and the appointment. Third, to the best of our knowledge, our study is the first in which the education was administered directly by the physician whom the patients visited; the same physician also reviewed their medical history and scheduled their colonoscopy. We believe that instructions provided by a physician whom the patients trust heighten the effect of the education. Fourth, the counseling emphasized on more than just simple dietary and purgative instructions; it stressed that poor bowel preparation could lead to missed lesions and that good bowel preparation could help in cancer prevention.

However, we acknowledge that the study has certain limitations as well. First, the sample size was small, and patient selection was not randomized. Because there was an age difference between the 2 groups, which may be a confounding factor, the data need to be further analyzed by means of a logistic regression model. Second, the indication for screening or surveillance was different in the 2 groups, making it difficult to identify whether the indications for colonoscopy were equally represented. Patients tend to be more aware of and adherent to educational information on bowel preparation when they have symptoms strongly suggestive of colon cancer.

In conclusion, physician-delivered education consisting of a brief counseling session followed by written instructions improves the quality of bowel preparation in outpatients undergoing screening colonoscopy. The instructions should emphasize on the proper diet before colonoscopy, adequate hydration after drinking the purgative, proper time for drinking the purgative, and importance of and rationale for thorough bowel preparation.

## Figures and Tables

**Table 1:** Baseline characteristics of the study patients and the effect of education on the outcome of bowel preparation and colonoscopy.

	Education		*P* value
	Yes (*n* = 39)	No (*n* = 60)	
Age (mean ± SD)	46.1 ± 10.9	52.8 ± 14.3	0.014*
Gender (male %)	25 (64.1%)	37 (61.7%)	0.81
Intubation time (minutes)	8.7 ± 4.4	9.2 ± 5.9	0.66
BBPS ≥ 5	38 (97.4%)	48 (80%)	0.01
Total score	7.3 ± 1.4	6.4 ± 1.9	0.012*
Right score	1.9 ± 0.8	1.7 ± 0.8	0.18
Transverse score	2.6 ± 0.5	2.2 ± 0.8	0.001*
Left score	2.7 ± 0.4	2.4 ± 0.6	0.006*
Adenoma detection rate	15/39 (38.5%)	13/60 (22.0%)	0.07
Adenoma detection number	0.72 ± 1.17	0.27 ± 0.55	0.029

Descriptive statistics for continuous data were calculated and were reported as mean ± standard deviation. Categorical variables were described using frequency distributions and were reported as *n* (%). Baseline characteristics and assessment results for the education group and controls were evaluated using Student's *t*-test for continuous variables and chi-square test for categorical variables. *P value < 0.05 was considered statistically significant.

## References

[1] Zauber AG, Winawer SJ, O’Brien MJ (2012). Colonoscopic polypectomy and long-term prevention of colorectal-cancer deaths. *The New England Journal of Medicine*.

[2] Ness RM, Manam R, Hoen H, Chalasani N (2001). Predictors of inadequate bowel preparation for colonoscopy. *American Journal of Gastroenterology*.

[3] Chokshi RV, Hovis CE, Hollander T, Early DS, Wang JS (2012). Prevalence of missed adenomas in patients with inadequate bowel preparation on screening colonoscopy. *Gastrointestinal Endoscopy*.

[4] van Rijn JC, Reitsma JB, Stoker J, Bossuyt PM, van Deventer SJ, Dekker E (2006). Polyp miss rate determined by tandem colonoscopy: a systematic review. *American Journal of Gastroenterology*.

[5] Rex DK, Imperiale TF, Latinovich DR, Bratcher LL (2002). Impact of bowel preparation on efficiency and cost of colonoscopy. *American Journal of Gastroenterology*.

[6] Madsen SM, Schlichting P, Davidsen B (2001). A patient education program is cost-effective for preventing failure of endoscopic procedures in a gastroenterology department. *American Journal of Gastroenterology*.

[7] Park JS, Sohn CI, Hwang SJ (2007). Quality and effect of single dose versus split dose of polyethylene glycol bowel preparation for early-morning colonoscopy. *Endoscopy*.

[8] Siddiqui AA, Yang K, Spechler SJ (2009). Duration of the interval between the completion of bowel preparation and the start of colonoscopy predicts bowel-preparation quality. *Gastrointestinal Endoscopy*.

[9] Chan W-K, Saravanan A, Manikam J, Goh K-L, Mahadeva S (2011). Appointment waiting times and education level influence the quality of bowel preparation in adult patients undergoing colonoscopy. *BMC Gastroenterology*.

[10] Froehlich F, Wietlisbach V, Gonvers J-J, Burnand B, Vader J-P (2005). Impact of colonic cleansing on quality and diagnostic yield of colonoscopy: The European Panel of Appropriateness of Gastrointestinal Endoscopy European multicenter study. *Gastrointestinal Endoscopy*.

[11] Seo EH, Kim TO, Park MJ (2012). Optimal preparation-to-colonoscopy interval in split-dose PEG bowel preparation determines satisfactory bowel preparation quality: an observational prospective study. *Gastrointestinal Endoscopy*.

[12] Lai EJ, Calderwood AH, Doros G, Fix OK, Jacobson BC (2009). The Boston bowel preparation scale: a valid and reliable instrument for colonoscopy-oriented research. *Gastrointestinal Endoscopy*.

[13] Hillyer GC, Basch CH, Basch CE (2012). Gastroenterologists’ perceived barriers to optimal pre-colonoscopy bowel preparation: results of a national survey. *Journal of Cancer Education*.

[14] Abbott SA (1998). The benefits of patient education. *Gastroenterology Nursing*.

[15] Griffin JM, Hulbert EM, Vernon SW (2011). Improving endoscopy completion: effectiveness of an interactive voice response system. *American Journal of Managed Care*.

[16] Liu X, Luo H, Zhang L (2013). Telephone-based re-education on the day before colonoscopy improves the quality of bowel preparation and the polyp detection rate: a prospective, colonoscopist-blinded, randomised, controlled study. *Gut*.

[17] Shaikh AA, Hussain SM, Rahn S, Desilets DJ (2010). Effect of an educational pamphlet on colon cancer screening: a randomized, prospective trial. *European Journal of Gastroenterology and Hepatology*.

[18] Spiegel BMR, Talley J, Shekelle P (2011). Development and validation of a novel patient educational booklet to enhance colonoscopy preparation. *American Journal of Gastroenterology*.

[19] Tae JW, Lee JC, Hong SJ (2012). Impact of patient education with cartoon visual aids on the quality of bowel preparation for colonoscopy. *Gastrointestinal Endoscopy*.

[20] Calderwood AH, Lai EJ, Fix OK, Jacobson BC (2011). An endoscopist-blinded, randomized, controlled trial of a simple visual aid to improve bowel preparation for screening colonoscopy. *Gastrointestinal Endoscopy*.

[21] Modi C, DePasquale JR, DiGiacomo WS (2009). Impact of patient education on quality of bowel preparation in outpatient colonoscopies. *Quality in Primary Care*.

[22] Hassan C, Bretthauer M, Kaminski MF (2013). Bowel preparation for colonoscopy: European Society of Gastrointestinal Endoscopy (ESGE) guideline. *Endoscopy*.

[23] Adler A, Wegscheider K, Lieberman D (2013). Factors determining the quality of screening colonoscopy: a prospective study on adenoma detection rates, from 12,134 examinations (Berlin colonoscopy project 3, BECOP-3). *Gut*.

[24] Belsey J, Crosta C, Epstein O (2012). Meta-analysis: the relative efficacy of oral bowel preparations for colonoscopy 1985–2010. *Alimentary Pharmacology and Therapeutics*.

